# Anti-apoptotic BCL-2 family proteins in acute neural injury

**DOI:** 10.3389/fncel.2014.00281

**Published:** 2014-09-30

**Authors:** Ujval Anilkumar, Jochen H. M. Prehn

**Affiliations:** Department of Physiology and Medical Physics, Centre for the Study of Neurological Disorders, Royal College of Surgeons in IrelandDublin, Ireland

**Keywords:** BCL-2, apoptosis, mitochondria, neuronal injury, neuronal development, neurodegeneration, ischemia, excitotoxcity

## Abstract

Cells under stress activate cell survival and cell death signaling pathways. Cell death signaling frequently converges on mitochondria, a process that is controlled by the activities of pro- and anti-apoptotic B-cell lymphoma 2 (BCL-2) proteins. In this review, we summarize current knowledge on the control of neuronal survival, development and injury by anti-apoptotic BCL-2 family proteins. We discuss overlapping and differential effects of the individual family members BCL-2, BCL-extra long (BCL-X_L_), myeloid cell leukemia 1 (MCL-1), and BCL2-like 2 (BCL-W) in the control of survival during development and pathophysiological processes such as trophic factor withdrawal, ischemic injury, excitotoxicity, oxidative stress and energy stress. Finally we discuss recent evidence that several anti-apoptotic BCL-2 proteins influence mitochondrial bioenergetics and control neuronal Ca^2+^ homeostasis independent of their classical role in cell death signaling.

## The BCL-2 protein family

The BCL-2 gene was first identified in B-cell follicular lymphomas (Tsujimoto et al., 1985). The BCL-2 protein family act as key regulators in the intrinsic or “mitochondrial” apoptosis pathway. The different BCL-2 protein family either trigger or constrain apoptosis (Youle and Strasser, 2008). They are classified into three different classes depending on their structural and functional properties: (a) anti-apoptotic BCL-2 proteins including BCL-2 itself, BCL-X_L_ (BCL-extra long), MCL-1 and BCL-W which contain four BH(1-4) domains (Czabotar et al., 2014); (b) pro pro-apoptotic proteins BAX (BCL-2-associated × protein), BAK (BCL-2-antagonist/killer-1) (Wei et al., 2001) and potentially BOK (BCL-2 related ovarian killer) that contain three conserved BH domains and interacts strongly with some anti-apoptotic proteins (Hsu et al., 1997); and (c) BH3-only proteins including BIM (BCL-2 interacting mediator), PUMA (p53 upregulated modulator of apoptosis), BID (BH3 interacting domain death agonist), BIK (BCL-2 interacting killer), BAD (BCL-2 associated death promoter), BMF (BCL-2 modifying factor, Hrk (Hara-kiri) and NOXA (Latin for “damage”) that have homology to the BCL-2 family proteins in only a single domain, the BH3 domain (Giam et al., 2008; Happo et al., 2012). While anti-apoptotic BCL-2 family proteins as well as BAX and BAK are often constitutively expressed in cells, BH3-Only proteins are typically transcriptionally or post-translationally activated in response to apoptotic stress signaling (Engel et al., 2011). Activation of the mitochondrial apoptosis pathway through pro-apoptotic BCL-2 proteins is able to activate different cell death pathways including apoptosis (Kilbride and Prehn, 2013). The key upstream event that leads to the activation of these different pathways is mitochondrial outer membrane permeabilization (MOMP). This process is triggered by the membrane insertion and oligomerization of the pro-apoptotic members BAX and BAK, with subsequent release of apoptosis-activating factors such as cytochrome c (cyt c) from the mitochondrial intermembrane space to the cytosol. Two models have been proposed for the activation of BAX and BAK during apoptosis: (a) the direct activation model where BAX and BAK activation occurs directly through conformational changes induced by BH3-only proteins (Letai et al., 2002) and (b) indirect activation model where cell death signals induce the binding of BH3-only pro-apoptotic initiators to anti-apoptotic BCL-2 proteins, facilitating the release and activation of BAX and BAK (Uren et al., 2007; Willis et al., 2007). Anti-apoptotic BCL-2 proteins are integral membrane proteins, possessing a C-terminal transmembrane domain that localizes these proteins to intracellular membranes, notably the mitochondrial outer membrane (MOM), but also the endoplasmic reticulum and the nuclear envelope (Cory and Adams, 2002; Chipuk et al., 2006; Brunelle and Letai, 2009; Tait and Green, 2010) and where they are also able to inhibit the process of MOMP by binding to pro-apoptotic BCL-2 proteins (Gonzalez-Garcia et al., 1994; Yang et al., 1995, 1997).

## Acute neuronal injury and BCL-2 proteins

Neurons are highly specialized, excitable cells that communicate to other target neurons through the process of synaptic transmission. Glutamate is the principal excitatory neurotransmitter in the CNS. Glutamate release from presynaptic nerve terminals activates post-synaptic glutamate receptors including NMDA, AMPA, and Kainate receptors (Fykse and Fonnum, 1996). However, overactivation of glutamate receptors can be neurotoxic, a process termed excitotoxicity (Olney et al., 1972). Excitotoxic neuronal cell death is primarily mediated by excessive Ca^2+^ influx via NMDA receptors (Choi, 1985, 1987, 1988) and has been implicated in neurological disorders including stroke, traumatic brain injury, ischemia, Huntington's disease and amyotrophic lateral sclerosis (Dirnagl et al., 1999; Waggie et al., 1999; Mehta et al., 2013). Glutamate neurotoxicity but also other processes such as ischemia/reperfusion injury also invoke oxidative stress (Barnham et al., 2004).

Recent studies have shown that excitotoxic and oxidative stress-induced neuronal injury involves BCL-2 family proteins. Specifically, injury conditions that produce a more delayed neuronal injury are often triggered by the transcriptional and post-translational activation of BH-3 only proteins such as BIM, PUMA, and BID (Konig et al., 2007; Steckley et al., 2007; Concannon et al., 2010). Similarly, the pro-apoptotic BAX protein has been shown to be implicated in excitotoxicity, oxidative stress and trophic factor deprivation-induced neuronal apoptosis (Deckwerth et al., 1996; D'Orsi et al., 2012). In this review we focus on recent advancements in describing the role of individual anti-apoptotic BCL-2 family proteins during neuronal development, injury and neurodegeneration.

## BCL-2

BCL-2 is widely expressed in the developing brain including, neuroepithelial cells of the ventricular zones as well as the post-mitotic cells of the cortical plate, cerebellum, hippocampus and spinal cord (Merry et al., 1994). BCL-2 knockout mice show normal embryonic development but present with lymphoid apoptosis, neuronal and intestinal lesions and terminal kidney disease (Veis et al., 1993). High expression of *BCL-2* mRNA was observed in the developing nervous system and reduced significantly in the post-natal brain (Abe-Dohmae et al., 1993; Merry et al., 1994). Interestingly, high level of BCL-2 expression was maintained in sensory and sympathetic adult neurons (Merry et al., 1994). BCL-2 acts as an important regulator of cell death in developing sympathetic neurons after neuronal growth factor deprivation, whereas BCL-2 is not involved in the survival of mature sympathetic neurons (Greenlund et al., 1995). Overexpression of BCL-2 inhibited BAX-mediated cytochrome-c release, caspase activation and cell death in nerve growth factor-deprived sympathetic neurons (Putcha et al., 1999). These results taken together indicate that BCL-2 plays an important role specifically during development of the nervous system.

Functional studies in primary neuron cultures and animal models indicated that BCL-2 overexpression protected hippocampal neurons against glutamate-mediated excitotoxicity, and significantly reduced lesion size in the hippocampus resulting from NMDA induced excitotoxic damage (Wong et al., 2005). Overexpression of BCL-2 blocked translocation of apoptosis inducing factor (AIF) from mitochondria to the nucleus, resulting in improved cortical neuron survival following focal cerebral ischemia (Zhao et al., 2004). BCL-2 deficient mice show enhanced oxidative stress and alterations in antioxidants in the brain (Hochman et al., 1998) and up-regulation of BCL-2 may aid DNA repair following oxidative stress (Deng et al., 1999). Interestingly, BCL-2 expression also inhibited apoptosis of newborn neurons following MCAO in adult rat brains (Zhang et al., 2006). Transgenic mice overexpressing BCL-2 in neurons resulted in hypertrophy of the nervous system caused by reduced naturally occurring cell death but also showed a 50% reduction in brain infarct volume compared to wild type mice after permanent ischemia induced by MCAO(Martinou et al., 1994). Furthermore, transplantation of embryonic stem cells overexpressing BCL-2 into the post-infarct brain cavity of adult rats after MCAO resulted in neuronal differentiation and improvements in functional recovery and behavioral testing (Wei et al., 2005). Of note, alterations in endoplasmic reticulum Ca^2+^ homeostasis have been shown to induce apoptosis in neurons (Mattson et al., 2000). BCL-2 also modulates ER Ca^2+^ content by decreasing ER Ca^2+^ uptake (Ferrari et al., 2002; Rudner et al., 2002) which supports axon regeneration and neurite outgrowth during energy stress and mobilizes intracellular calcium signaling (Jiao et al., 2005). These results suggest that BCL-2 may represent an interesting target in stroke recovery therapy.

## BCL-X_L_

The BCL-X gene can be alternatively spliced to produce two protein isoforms, BCL-X_L_ and BCL-X_S_ (Gonzalez-Garcia et al., 1994). BCL-X_L_ acts as an anti-apoptotic protein whereas BCL-Xs exhibits pro-apoptotic properties. BCL-X_L_ is found in post-mitotic cells in the adult brain whereas BCL-X_S_ expression is predominantly expressed in developing cells with a high turnover rate such as lymphocytes (Boise et al., 1993). BCL-X_L_ is highly expressed in developing neurons as they migrate away from ventricular zone, and remains up-regulated in post-mitotic neurons in the adult brain (Motoyama et al., 1995; Roth et al., 2000). BCL-X_L_ shows close homology to BCL-2 (Gonzalez-Garcia et al., 1994). Deletion of BCL-X induces massive apoptotic cell death in developing neurons throughout the nervous system and results in lethality at embryonic day 13 (Motoyama et al., 1995; Akhtar et al., 2004). BCL-X_L_ also protects cultured sympathetic neurons against nerve growth factor withdrawal (Gonzalez-Garcia et al., 1995). These data suggest that BCL-X_L_ plays important roles during development of the nervous system; in addition BCL- X_L_ is also expressed at high levels in the adult nervous system.

Overexpression of BCL-X_L_ protected neurons in the hippocampus and cortex against hypoxic-ischemia (Parsadanian et al., 1998). Systemic delivery of BCL-X_L_ fusion protein inhibited caspase-3 and -9 activities and also prevented translocation of AIF into the nucleus following hypoxic-ischemic brain injury (Yin et al., 2006). Interestingly, ischemic preconditioning blocked the assembly of BAD with BCL-X_L_, cleavage of BCL-X_L_ to a pro-apoptotic form, and release of pro-apoptotic factors from mitochondria (Miyawaki et al., 2008). Overexpression of BCL-X_L_ also protected primary rat septal neurons against oxygen glucose deprivation and hypoglycaemic stress (Panickar et al., 2005). Furthermore, decreased expression of BCL-X_L_ has been implicated in spinal cord injury induced neuronal cell death. This was attenuated by exogenous administration of a BCL-X_L_ fusion protein into the spinal cord (Nesic-Taylor et al., 2005). In addition, transplantation of neural stem cells overexpressing BCL-X_L_ enhanced graft survival by supplying tropic factors essential for survival, and improved locomotor recovery in rats following spinal cord injury (Lee et al., 2009).

BCL-X_L_ is also involved in non-apoptotic processes such as synapse formation. Overexpression of BCL-X_L_ in hippocampal neurons increased synapse numbers and localization of mitochondria to synapses, a process that was modulated through the mitochondrial fission protein, dynamin related protein 1 (Li et al., 2008). Interestingly, BCL-X_L_ demonstrated a dual role in synaptic transmission under hypoxia (Hickman et al., 2008). Inhibition of BCL-X_L_ resulted in reduced recovery of synaptic responses under hypoxia, but exerted neuroprotective effect (Hickman et al., 2008). Recently, it has been demonstrated that BCL-X_L_ is important in maintaining mitochondrial fission, fusion and biomass (Berman et al., 2009), and directly interacts with ATP synthase to stabilize the mitochondrial membrane potential (Chen et al., 2011). BCL-X_L_ has also been demonstrated to influence Ca^2+^ signaling in astrocytes induced by the activation of inositol 1,4,5-triphosphate (IP_3_)-generating metabotropic type 5 glutamate receptors (mGluR5) during the process of motoneuron degeneration. Administration of the BH4 domain of BCL-X(L) fused to the protein transduction domain of the HIV-1 TAT protein was sufficient to restore Ca^2+^ homeostasis in astrocytes overexpressing the ALS-associated SOD1 (G93A) mutation, and chronic treatment of SOD1(G93A) transgenic mice with the TAT-BH4 peptide delayed the onset of the disease and improved motor function and lifespan (Martorana et al., 2012).

## BCL-w

*BCL-w* also known as BCL2-like 2 (Bcl2l2) is a highly conserved gene located on the mouse chromosome 14 and the human chromosome 14 at band q11. BCL-w is highly expressed particularly in the brain, colon and testes and is also associated with intracellular membranes (O'Reilly et al., 2001). Although BCL-w is widely expressed, mice deficient in BCL-w failed to show large abnormalities with the exception of increased apoptosis in sperm cells during spermatogenesis, resulting in a sterile male phenotype (Print et al., 1998). The level of BCL-w has been shown to increase during neuronal development and BCL-w has been localized to specific regions of the mature brain where it may play a crucial role in maintaining their neuronal survival (Hamner et al., 1999). In this context, BCL-w overexpression increased neuronal survival in NGF-dependent trigerminal neurons and BDNF- dependent nodose neurons over the period of development in response to neurotrophin withdrawal (Middleton et al., 2001).

**Table 1:** **Non-cell death related functions of anti-apoptotic proteins**.

**Anti-apoptotic proteins**	**Physiological state**
BCL-2	Regulates ER Ca^2+^ homeostasis by decreasing the ER Ca^2+^ uptake (Ferrari et al., 2002; Rudner et al., 2002)
	Supports axon regeneration and neurite outgrowth (Jiao et al., 2005)
BCL-X_L_	Involved in synapse formation
	Increases synapse number and localization of mitochondria to synapse (Hickman et al., 2008)
	Maintains mitochondrial fusion, fission, and biomass (Berman et al., 2009)
	Stabilizes mitochondrial membrane potential by directly interacting with ATP synthase (Chen et al., 2011)
	Stabilizes IP_3_-receptor mediated Ca^2+^ signaling in astrocytes (Martorana et al., 2012)
BCL-w	Regulates neuronal excitability by modulating GABA-mediated currents (Murphy et al., 2007)
MCL-1	Localizes on mitochondrial outer membrane and inner membrane. Antagonizes anti-apoptotic proteins and maintains normal mitochondrial bioenergetics status (Perciavalle et al., 2012)
	Regulates mitochondrial fusion, fission, and cristae formation and facilitates ATP production (Perciavalle et al., 2012)
	Maintains cystosolic Ca^2+^ homeostasis and increases mitochondrial membrane potential (Anilkumar et al., 2013)

Functional studies revealed that BCL-w interacts with the pro-apoptotic protein BAD and blocks neuronal death induced by growth factor deprivation in sympathetic neurons (Hamner et al., 2001). Interestingly, BCL-w expression was found to be increased in mouse neurons up to 72 h after transient middle cerebral artery occlusion (MCAO). Moreover, BCL-w co-localized with mitochondria in non-fragmented neurons and protected neurons against Ca^2+^ mediated brain injury by inhibiting cytochrome-c release and maintaining mitochondrial membrane potential following MCAO (Yan et al., 2000). Mouse deficient in BCL-w showed an increased neuronal loss and nuclear fragmentation in the hippocampus after status epilepticus with a neurophysiological phenotype leading to earlier onset of seizure, and was suggested to influence neuronal excitability (Murphy et al., 2007). This effect may be regulated through an effect of BCL-w on GABA-mediated currents, a disruption of which may lead to seizure induction (Murphy et al., 2007). Furthermore, in an experimental model of Alzheimer's disease, BCL-w blocked the mitochondrial release of Smac and inhibited neuronal apoptosis induced by β-amyloid (Yao et al., 2005). Using a rat model of transient MCAO and oxygen glucose deprivation in neurons, it was demonstrated that BCL-w plays an important role in neuroprotection following ischemic injury and is directly regulated by microRNA-29b (Shi et al., 2012). There results taken together suggest that BCL-w plays an important role in various neurological conditions to protect neurons and therefore also presents an attractive target to development of therapeutic agents.

## MCL-1

The human *MCL-1* gene is located on chromosome 1q21, and MCL-1 proteins were originally isolated from myeloid leukemia cells (Kozopas et al., 1993). MCL-1 is prominently expressed in neuroendocrine cells, sympathetic neurons, cardiac and skeletal muscles (Krajewski et al., 1995). Importantly, diffuse expression and rapid induction of transcription of *MCL-1* is found in neurons (Mori et al., 2004). MCL-1 germline deletion in mice resulted in peri-implantation lethality at embryonic day 3.5, showing the most severe phenotype amongst the anti-apoptotic BCL-2 family members (Rinkenberger et al., 2000). MCL-1 is associated with membranes through its C-terminal hydrophobic tail and has a predominant mitochondrial localization (Yang et al., 1995). Recently, it has been demonstrated that MCL-1 is spliced into two variants and resides in two distinct mitochondrial regions: outer membrane (MCL-1^OM^) and matrix (MCL-1^MATRIX^), and has been proposed to exert differential effects depending on its location: MCL-1^OM^ antagonizes apoptosis, whereas MCL-1^MATRIX^ facilitates ATP production, membrane potential, respiration, cristae ultrastructure and mitochondrial fusion (Perciavalle et al., 2012).

MCL-1 is essential for neuronal development. Conditional deletion of MCL-1 in mice induced apoptosis of neuronal progenitors and newly committed neurons as they commence their migration away from the ventricular zone (Arbour et al., 2008). In addition, conditional deletion of MCL-1 *in vitro* in neuronal precursor cells showed a two-fold increase in apoptosis (Malone et al., 2012). These results suggested that MCL-1 is crucial for survival of neuronal precursor cells. Recently, it has been demonstrated that MCL-1 can act as a switch between autophagy and apoptosis in a developmentally regulated manner under energetic stress conditions (Germain et al., 2011). Being an anti-apoptotic BCL-2 family member, MCL-1 inhibited apoptosis by binding and sequestering the pro-apoptotic BCL-2 family member BAK (Willis et al., 2005) and blocking the activation and translocation of BAX (Chen et al., 2007). Interestingly, MCL-1 protected cortical neurons against NMDA-mediated excitotoxicity, but also increased mitochondrial bioenergetics of cortical neurons and normalized neuronal Ca^2+^ homeostasis during NMDA excitation (Anilkumar et al., 2013). Such dual effects of MCL-1 may make it an attractive target for the treatment of neurodegenerative disorders such as Alzheimer's and Parkinson's disease, in which altered bioenergetics and increased neuronal loss are so prominent. MCL-1 heterozygous mice also demonstrated increased neuronal sensitivitiy against pilocarpine-induced status epilepticus (Mori et al., 2004). Furthermore, sustained expression of MCL-1 protected and loss of MCL-1 increased DNA damage- induced neuron death (Arbour et al., 2008). A recent study has also shown that apoptosis induced by serum and KCl deprivation in cerebellar granule neuron was mediated by proteasomal degradation of MCL-1, and that stabilization of MCL-1 by blocking its ubiquitination and degradation was protective (Magiera et al., 2013). In addition, neurons depleted of Parkin, mutated gene in juvenile onset and familial forms of Parkinson's disease, became acutely sensitive to oxidative stress, and this was attributed to decreased MCL-1 levels (Ekholm-Reed et al., 2013). Although the role of MCL-1 in neurons and neurodegenrative disorders warrants further investigation, emerging evidence suggests that it may represent the most promising therapeutic target of all BCL-2 family proteins studied so far.

## Conclusion

BCL-2 family proteins have been firmly established to play a significant role in initiating or inhibiting apoptosis during neuronal development and injury. Emerging evidence suggests that some of the anti-apoptotic protein family members are involved in maintaining both mitochondrial bioenergetics and neuronal survival, in particular BCL-X_L_ and MCL-1. BCL-2, BCL-X_L_ and MCL-1 modulation of Ca^2+^ signaling during acute neuronal injury may also play a vital role in neuroprotection. Hence manipulating the pro-survival BCL-2 family members may be beneficial in developing future therapies for neurological and neurodegenerative disorders. In addition, exploiting neural stem cells overexpressing anti-apoptotic BCL-2 family proteins may provide an attractive and powerful tool for post-neuronal injury therapies.

### Conflict of interest statement

The authors declare that the research was conducted in the absence of any commercial or financial relationships that could be construed as a potential conflict of interest.
